# Habitual Physical Activity of People with or at Risk of Diabetes-Related Foot Complications

**DOI:** 10.3390/s23135822

**Published:** 2023-06-22

**Authors:** Byron M. Perrin, Dimitri Diacogiorgis, Courtney Sullivan, James Gerrard, Isabelle Skinner, Timothy C. Skinner, Rashmika Nawaratne, Damminda Alahakoon, Michael I. C. Kingsley

**Affiliations:** 1 La Trobe Rural Health School, La Trobe University, Bendigo 3552, Australia; 2 Holsworth Research Initiative, La Trobe University, Bendigo 3550, Australia; 3 Grampians Health Ballarat, Ballarat 3350, Australia; 4 Central Australian Aboriginal Congress, Mparntwe (Alice Springs) 0870, Australia; 5 School of Psychology and Public Health, La Trobe University, Bendigo 3552, Australia; 6 Research Centre for Data Analytics and Cognition, La Trobe University, Bundoora 3086, Australia; 7 Department of Exercise Sciences, University of Auckland, Auckland 1023, New Zealand

**Keywords:** diabetes, diabetic foot, peripheral neuropathy, physical activity, exercise

## Abstract

Regular physical activity is an important component of diabetes management. However, there are limited data on the habitual physical activity of people with or at risk of diabetes-related foot complications. The aim of this study was to describe the habitual physical activity of people with or at risk of diabetes-related foot complications in regional Australia. Twenty-three participants with diabetes from regional Australia were recruited with twenty-two participants included in subsequent analyses: no history of ulcer (N = 11) and history of ulcer (N = 11). Each participant wore a triaxial accelerometer (GT3X+; ActiGraph LLC, Pensacola, FL, USA) on their non-dominant wrist for 14 days. There were no significant differences between groups according to both participant characteristics and physical activity outcomes. Median minutes per day of moderate-to-vigorous physical activity (MVPA) were 9.7 (IQR: 1.6–15.7) while participants recorded an average of 280 ± 78 min of low-intensity physical activity and 689 ± 114 min of sedentary behaviour. The sample accumulated on average 30 min of slow walking and 2 min of fast walking per day, respectively. Overall, participants spent very little time performing MVPA and were largely sedentary. It is important that strategies are put in place for people with or at risk of diabetes-related foot complications in order that they increase their physical activity significantly in accordance with established guidelines.

## 1. Introduction

People with diabetes are at risk of developing lower-extremity complications, such as peripheral neuropathy and peripheral artery disease, which can lead to foot ulceration and lower-extremity amputation [1]. Diabetes-related foot complications are a large and growing contributor to the disability burden worldwide, globally accounting for an estimated 59% of all diabetes-related years lived with disability [2]. Diabetes-related foot complications are hard to manage and often recur [3], negatively influence quality of life [4], and are disproportionately represented in socially disadvantaged populations and in regional and rural geographic areas [5,6]. 

The American Diabetes Association guidelines recommend that adults with diabetes should engage in 150 min or more of moderate-to-vigorous physical activity per week [7]. People who participate in diabetes lifestyle interventions that increase physical activity can reduce reliance on medications through a range of metabolic benefits (e.g., body mass control, reduced blood pressure, enhanced insulin sensitivity, improved lipoprotein balance) as well as enhancing musculoskeletal function [8]. The positive effects of exercise training on glucose control, physical function, and the signs and symptoms of peripheral neuropathy have been confirmed via meta-analyses of studies on people with existing neuropathy [9]. Furthermore, with adequate baseline screening and participant selection adverse events are unlikely, and the risk of further ulceration in response to exercise is low [9,10,11]. 

Early research investigating daily activity patterns in patients with diabetic foot complications indicated that people with diabetes and peripheral neuropathy take fewer steps, but with more variation in step counts (or “spikes”), and that more steps are taken indoors than outdoors [12,13]. One Australian group has reported that people with current active diabetes-related ulceration take fewer steps but expend more energy than those without ulceration [14]. People with diabetes have been reported to undertake more steps indoors than outdoors, but the authors of a recent review concluded that future research using technology to investigate a variety of outcomes related to physical activity such as standing/sedentary time and bouts of activity in different settings is needed [15]. 

It is currently unclear if people with or at risk of diabetes-related foot complications in regional and rural populations in Australia are meeting physical activity guidelines. A better understanding of the habitual activity patterns in this population has the potential to inform effective activity-based preventative health interventions for both primary and secondary prevention that could reduce the health burden of people with diabetes in regional and rural Australia. Thus, the aim of this study was to describe the habitual physical activity of people with or at risk of diabetes-related foot complications in regional Australia.

## 2. Materials and Methods

This observational study recruited participants from a high-risk foot service in regional Australia. Inclusion criteria were diagnosis of type 2 diabetes. Exclusion criteria were less than 18 years old, an active skin ulceration on the foot, and inability to provide informed consent. Potentially eligible participants from a high-risk foot service in regional Victoria were invited to participate in the study. The podiatry-led high-risk foot service is a multi-disciplinary service and accepts referrals for people with diabetes across the entire spectrum of diabetes-related foot disease. Referral and prioritising systems in place ensure people at high risk of or with active foot morbidity are managed by the tertiary high-risk foot service, with maintenance and prevention services undertaken by affiliated community health services. Ethical clearance was provided by institution ethics committees (LNR/14/BHSSJOG/24), and informed consent was provided by all participants.

General participant characteristics were measured and included age, sex, body mass index (BMI), diabetes history (diabetes type, diabetes duration, and method of diabetes control), and foot morbidity (foot deformity, presence of peripheral neuropathy, history of ulceration, and history of amputation). To measure physical activity outcomes, each participant wore a triaxial accelerometer (GT3X+; ActiGraph LLC, Pensacola, FL, USA) calibrated and synchronised to record triaxial accelerations at 100 Hz on their non-dominant wrist for 14 days, 24 h per day, except when they were likely to submerge the accelerometer underwater. ActiLife software (version 7.0; ActiLife Corp., Pensacola, FL, USA) was used to obtain the raw triaxial acceleration data, which was downloaded in epoch lengths of 60 s. Non-wear time was determined using the Choi wear time validation algorithm [16], while sleep time was differentiated from wake time using the Cole–Kripke sleep algorithm [17]. Periods classified as non-wear and/or sleep were removed from the dataset prior to the export of raw triaxial acceleration data. To be included in the analysis, two valid weeks of data were required where a valid week was defined as ≥10 h per day of wear time on at least five days including one weekend day [18].

In line with recent research demonstrating the unfavourable performance of linear regression-based predictive models and traditional machine learning-based predictive models to estimate the intensity of physical activity in wrist-worn accelerometers in free-living conditions [19], a Convolutional Neural Network (CNN) variant of a deep learning algorithm was used to predict energy expenditure and the intensity of physical activity [20]. The CNN deep learning method has been shown to closely predict activity intensity (83.7%, 95% CI: 80.9–86.5%) and has recently demonstrated a strong correlation with energy expenditure predictions (r = 0.86, 95% CI: 0.84–0.87) with a reference hip-specific method (modified Freedson VM3 Combination equation) [20]. Daily physical activity outcomes predicted by the CNN models were energy expenditure (kJ); average metabolic equivalents (METs); and minutes spent in sedentary activity, low-intensity physical activity (LPA), and moderate-to-vigorous physical activity (MVPA). Additionally, non-wear time (minutes) and the sleep-related measures of average sleep duration (minutes) and sleep efficiency (%) were also measured. 

In addition to these commonly reported measures of physical activity, MX metrics were also extracted. MX is a novel accelerometer metric that captures the intensity (acceleration) during a person’s most active period of the day [21]. MX metrics are translational metrics that facilitate meaningful public-health messages due to their ability to be described in terms of activities (e.g., fast walking) or intensity (e.g., moderate-to-vigorous physical activity). MX metrics were reported as the average magnitude of dynamic acceleration (corrected for gravity) averaged over 5 s epochs and expressed as milligravitational units (m*g*) [21,22]. MX metrics have previously been used to report physical activity and chronotype in people with type 2 diabetes [23]. The most active 480 min or third of a day (M480), 120 min (M120), 60 min (M60), 30 min (M30), 10 min (M10), 5 min (M5), and 2 min (M2) were recorded and compared to approximate accelerations associated with a slow (100 m*g*) and fast (200 m*g*) walk [20,21,24].

Signal processing to process multi-day raw accelerometer data for physical activity and sleep research included autocalibration using local gravity as a reference [25]; detection of sustained abnormally high values; detection of non-wear; and calculation of the average magnitude of dynamic acceleration, corrected for gravity averaged over 5 s epochs and expressed in milligravitational units (m*g*). Participants were excluded if their accelerometer files showed a post-calibration error greater than 0.01 g (10 m*g*), if they had fewer than 3 days of valid wear (defined as >16 h per day), or if wear data were not present for each 15 min period of the 24 h cycle [26,27]. The default non-wear setting was used whereby invalid data were imputed via the average at similar time-points on different days of the week. The average of all valid days was used for all outcome variables. 

For the CNN modelling, inputs for predictive model development were the raw 100 Hz accelerometer files extracted from ActiLife with models developed using Python programming language (Python Software Foundation, https://www.python.org/; accessed on 23 January 2023). To extract and visually represent MX metrics, the raw GT3X files exported from ActiLife were processed using the R-package GGIR (version 2.8–2; http://cran.r-project.org; accessed on 23 January 2023) [21]. 

Physical activity was analysed for the entire sample. As the risk for a future ulceration is extremely high for people with a previous ulceration, secondary between-groups analysis was undertaken for participants who had a history of diabetes-related foot ulceration compared those who had not. Final data analysis was undertaken using IBM SPSS Statistics for Windows (version 24.0; IBM Corp., Armonk, NY, USA). For continuous variables with normal distributions, means and standard deviations were reported with differences between groups assessed using independent samples’ *t*-tests. Continuous variables that did not conform to the assumptions of normal distributions were presented as medians and interquartile ranges (IQR), and differences between groups were assessed using the Mann–Whitney U-test. For categorical variables, proportions were reported, and differences between groups were tested using chi-squared with continuity correction. Statistical significance was set at *p* < 0.05. 

## 3. Results

Twenty-three participants were recruited for this study. One participant was excluded due to mechanical failure of the accelerometer. Therefore, 22 participants were included in subsequent analyses, with 11 participants having a history of ulceration and 11 who did not have a history of ulceration. 

There were no significant differences between those with history of ulcer when compared to those without a history of ulcer according to both participant characteristics (Table 1) and physical activity or sleep outcomes (Table 2).

**Figure 1:**
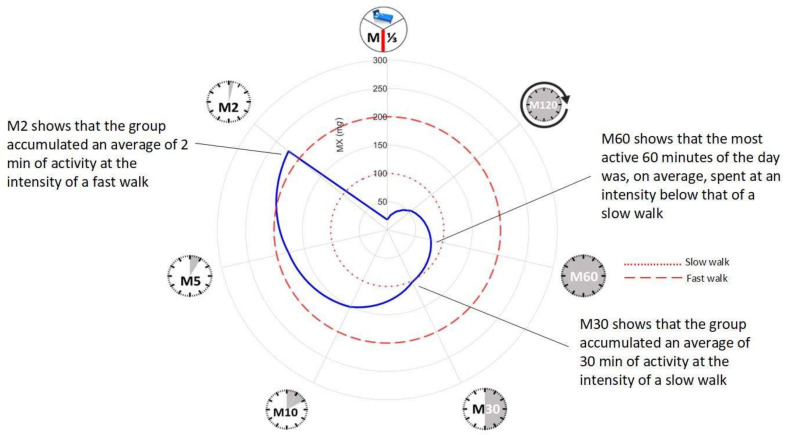
Radar plot illustrating mean MX metrics for the sample (clockwise) for the most active 8 h of the day (M1/3DAY), 120 min (M120), 60 min (M60), 30 min (M30), 10 min (M15), 5 min (M5), and 2 min (M2). The dashed red lines represent indicative values for: slow walking (........) and fast walking (---) [24].

The median accelerations of the most active continuous 2, 5, 10, 30, 60, 120, and 480 min were 223 m*g* (IQR 182-276 m*g*), 179 m*g* (IQR 146-223 m*g*), 151 m*g* (IQR 122–182 m*g*), 102 m*g* (IQR 89–125 m*g*), 79 m*g* (IQR 70–97 m*g*), 54 m*g* (IQR 50–71 m*g*), and 18 m*g* (IQR 16–23 m*g*), respectively. Figure 1 presents a radar plot illustrating continuous MX metrics. The dashed circles reflect accelerations that are associated with a slow (blue) and a fast (red) walk [22]. The sample achieved on average 30 min of slow walking and 2 min of fast walking per day (Figure 1).

## 4. Discussion

This study describes the habitual physical activity and sleep characteristics of people with or at risk of diabetes-related foot complications in a regional Australian population. The results showed that the participants were insufficiently active with high amounts of sedentary behaviours, and they slept for shorter durations than recommended.

The American Diabetes Association guidelines recommend that adults with diabetes should engage in 150 min or more of moderate-to-vigorous activity per week, undertake daily exercise (with no more than two consecutive days without activity) including both aerobic and resistance exercises, and reduce sedentary behaviour [7]. The participants in this study performed less than half the recommended weekly MVPA, mean METs indicated daily intensity of activity was equivalent only to sitting [28,29], and MX metrics indicated that that participants failed to engage in daily physical activity at an intensity above that of a slow walk for 60 continuous minutes. This in contrast to (pre COVID-19 pandemic) Australian census data showing that 26.1% of adults 65 years and over engaged in 30 min of exercise on 5 or more days [30]. These findings are concerning. Physical activity has many benefits for people with diabetes, including those with or at risk of diabetes-related foot complications; benefits include weight reduction, enhanced blood glucose control, reduced cardiovascular risk factors, improved balance, and likely also improved peripheral nerve function [7,9,31]. Additionally, high sedentary time has been found to be an independent predictor of the development of diabetes-related ulceration in people with peripheral neuropathy [32], and it is also associated with an increased risk for metabolic syndrome, cardiovascular disease, and all-cause mortality [33,34]. 

It is clear that there are positive health benefits of exercise for people with or at risk of diabetes-related foot complications [9,35]. There is also consolidated information about the recommended types of exercise, as well as frequency and duration. The American Diabetes Association position statement on exercise and type 2 diabetes recommends that all adults with type 2 diabetes reduce sedentary time and that a combination of aerobic and resistance exercise training is required for optimal glycaemic and health outcomes [7]. For most adults, this includes 150 min or more of moderate-to-vigorous activity weekly, and 2–3 sessions/week of resistance exercise [7]. The ADA also recommends supervised training over non-supervised training [7]. More recently, Streckman et al. published findings from meta-analyses investigating exercise interventions for people with diabetes-related peripheral neuropathy in order to derive evidence-based recommendations [9]. A total of 27 randomised controlled trials were included in the review, and results showed that aerobic training of at least moderate intensity (40–70% heart rate reserve) 3–6 times per week for 12 weeks improves glucose control and may improve peripheral nerve conduction. Sensorimotor training was also identified to improve static balance, playing a role in targeting balance control and sensory and motor signs and symptoms of peripheral neuropathy [9].

There are challenges to facilitating increased physical activity, and a variety of individual and social factors have been shown to influence physical activity uptake in adults such as age, income, rurality, and social support, and the relatively low physical activity and sleep duration reported in this study are consistent with non-urban, older populations [36,37,38]. These factors may contribute to the gap that exists between intent and actual behaviour, with as many as 46% of individuals failing to follow through with their long-term intentions regarding physical activity [39]. Data from this aforementioned meta-analysis indicate that motivation, self-regulation, and habit/automaticity are also likely to be influential psychological constructs [39]. Further challenges in the sub-population include a reluctance from health professionals to facilitate increased weight-bearing physical activity through a perceived concern of skin trauma and subsequent ulceration. Current national Australian recommendations from Diabetes Feet Australia do not provide information about exercise, apart from cautioning that an increase in physical activity should be gradual [40]. However, as early as 2008 evidence emerged that weight-bearing activity for this population does not increase the rate of foot ulcers, and there is a call for a paradigm shift towards maintaining and increasing physical activity [41,42]. Results from subsequent studies have consistently demonstrated that with adequate baseline screening, participant selection, and appropriate footwear, the risk of further ulceration in response to activity is low [9,10,11]. The European Wound Management Association has investigated the exercise interventions in people with current ulceration and suggests that some weightbearing is not detrimental to ulcer healing (if appropriate footwear is ensured) [43]. Better mechanisms are needed to assist health professionals and people with diabetes to increase their physical activity in accordance with guidelines and their particular circumstances. This should include access to affordable health professional support. In Australia, this could include better utilisation of government funding sources to enable engagement of an exercise physiologist for appropriate assessment and subsequent design of an exercise program, which can include group exercise programs [11,44].

Total MVPA time is lower than previously reported in a metropolitan Australian setting. Lee at al. reported that participants with diabetes with or at risk of diabetes-related foot morbidity performed ≥30 min of MVPA most days of the week, although the MVPA was accumulated in short durations rather than meeting the criteria of an exercise bout and therefore was likely to be incidental [45]. It is also possible that the discrepancy in findings can be attributed to differences in the micro-technology and algorithms used to estimate physical activity intensities [18,46]. The previous Australian research reported daily physical activity outcomes from data derived from the SenseWear Armband (BodyMedia Inc.; Pittsburgh, PA, USA) in a similar population to the current study [14,45], and caution is required when making comparisons [19]. The sedentary time of over eleven hours each day is similar to previous studies using accelerometry [14], and the self-reported Physical Activity Scale [32] data to measure physical activity. The MX metrics findings are comparable to those reported by authors of a relatively large observational trial of people with type 2 diabetes who also failed to meet or just met 60 min of continuous activity at or above an intensity reflective of a slow walk [23,47].

Recommended sleep duration for adults up to 64 years is 7–9 h per day, and for those over 64 years is 7–8 h per day [48]. The sample reported a shorter sleep duration than these recommendations, and shorter sleep duration than a representative sample from the general population of people aged over 64 years [49]. For people with diabetes, a short duration of sleep has been shown in a meta-analysis to be associated with worse glycaemic control [50]. Restricted sleep is thought to effect hormonal regulation of appetite, leading to elevated appetite that could lead to an increase in body mass index and insulin resistance [50,51], and a recent cohort study has shown that people with diabetes with inadequate sleep duration have a higher risk of coronary heart disease and all-cause mortality [52]. For a person with diabetes, reduced sleep can also impact the inclination to undertake self-care behaviours and exercise [53]. Specific data on sleep in people with or at risk of diabetes-related foot complications is limited, with a recent scoping review identifying 12 heterogeneous observational studies that investigated a variety of foot health and sleep outcomes [54]. The authors suggest a possible association between obstructive sleep apnea and the presence or history of diabetes-related foot ulceration, but high-quality research is needed to understand the role sleep duration and quality has on the prevention or treatment of diabetes-related foot complications.

Although the between-groups analysis did not find a statistically significant difference in physical activity or sleep outcomes between participants with no history of ulcer and those with history of ulcers, there was a trend for the history of ulcer (i.e., higher-risk) group to exhibit increased energy expenditure in comparison to the group with no ulcer history. Increased energy expenditure has also been observed in worse foot morbidity groups such as those with a current diabetes-related foot ulcer [14]. For those with an active ulcer or peripheral neuropathy, it is possible that increased energy expenditure is due to wound healing and the adoption of an inefficient gait pattern [14,55], but research into possible energy imbalance across people with different levels of foot morbidity is required [14]. 

The results of the study need to be interpreted considering some limitations. The sample size is small, and from a regional area of Australia. The findings might not be generalisable to other populations from different geographical areas, and the between-groups analysis may be underpowered to make definitive conclusions. The descriptive nature of the study did not allow detailed assessment of broad factors associated with undertaking physical activity or sleep patterns. However, this is the first time that data from a regional Australian population has been reported, which has extended previous Australian research [14,45]. Wear time from participants indicated excellent participant adherence to the 14-day data collection period of continual 24 h accelerometer wear, similar to the previous Australian studies [14,45]. This is consistent with emerging evidence that people with or at risk of diabetes-related foot complications have a positive attitude and high self-efficacy towards using technology to monitor foot health [56]. Wearable devices, such as accelerometer-based motion sensors, can capture a variety of physical activity outcomes and have gained popularity due to their ease of use, availability, and objective measurement of physical activity [46]. However, large variances can exist in predicted physical activity according to the type of accelerometer, where the device is worn (e.g., wrist or waist), and analysis technique [18,19,46]. To overcome some of these challenges, the ActiGraph triaxial accelerometer used in this study (GT3X+; ActiGraph LLC, Penascola, FL, USA) has been shown to be reliable and valid for older adults with type 2 diabetes [57], and to overcome the limitations of traditional linear-regression-based predictive analysis of physical activity, a CNN deep learning algorithm was used to estimate energy expenditure in this population [20]. The MX metrics, reported for the first time for this population, provide practical ways to conceptualise patterns of physical activity.

## 5. Conclusions

This is the first study of habitual physical activity patterns and sleep duration of people with or at risk of diabetes-related foot complications in a regional Australian population. A variety of novel validated measures of physical activity and sleep were used for this population. Participant engagement with the monitoring was excellent. By all measures, the results showed that people with or at risk of diabetes-related foot complications are not meeting physical activity or sleep duration guidelines, undertake insufficient MVPA, and are largely sedentary. The novel MX metric data suggest that the most active 60 min of each day are undertaken at an intensity lower than a slow walk. It is important that health professionals seek ways to facilitate significantly increased habitual physical activity in this population in accordance with established guidelines. 

## Figures and Tables

**Table 1:** Participant characteristics.

Characteristic	Total (*n* = 22)	History of Ulcer (*n* = 11)	No History of Ulcer (*n* = 11)	*p*-Value
Diabetes type				0.534
Type 1	3 (13.6%)	2 (18.2%)	1 (9.1%)	
Type 2	19 (86.4%)	9 (81.8%)	10 (90.9%)	
Male sex	12 (54.5%)	7 (63.6%)	5 (45.4%)	0.669
Age (years)	65 ± 10	61 ± 7	69 ± 11	0.076
Body mass index	33.0 ± 7.0	34.9 ± 8.2	30.7 ± 6.0	0.186
Diabetes duration (years)	18 ± 10	20 ± 9	15 ± 11	0.348
Previous amputation	3 (13.6%)	3 (27.3%)	0 (0.0%)	0.062
Presence of peripheral neuropathy	15 (68.2%)	11 (100%)	4 (36.4%)	0.001
Foot deformity	11 (50.0%)	7 (63.6%)	4 (36.4%)	0.394
Method of diabetes control				0.873
Insulin	12 (57.1%)	6 (54.5%)	6 (54.5%)	
Oral hypoglycaemics	7 (33.3%)	2 (18.2%)	5 (45.5%)	
Combination	2 (9.5%)	2 (18.2%)	0 (0.0%)	

Note: due to data collection error, the reporting of variables for diabetes duration and diabetes control is based on 21 participants only. Data presented as number (%) or mean ± SD.

**Table 2:** Daily physical activity and sleep outcomes.

Daily Activity Outcomes	Total (*n* = 22)	History of Ulcer (*n* = 11)	No History of Ulcer (*n* = 11)	*p*-Value
METs	1.5 ± 0.1	1.5 ± 0.1	1.4 ± 0.7	0.246
Energy expenditure (kJ) ^	14,262 (1164–18,797)	16,787 (11,465–20,707)	13,189 (11,700–14,539)	0.101
Sedentary behaviour (minutes)	689 ± 114	676 ± 105	702 ± 125	0.664
LPA (minutes)	280 ± 78	286 ± 82	274 ± 77	0.816
MVPA (minutes) ^	9.7 (1.6–15.7)	10.8 (1.6–15.8)	4.6 (1.1–15.7)	0.478
Sleep duration (hours)	5.6 ± 1.7	5.3 ± 1.5	6.0 ± 1.9	0.149
Sleep efficiency (%) *	92.7 ± 3.8	92.1 ± 4.7	93.3 ± 2.7	0.471
Accelerometer non-wear time (minutes) ^	96 (44–198)	105 (5–223)	74 (17–172)	0.236

Data reported mean ± SD unless otherwise stated. * Proportion of time asleep while in bed. ^ Median (interquartile range, IQR). MET, metabolic equivalent of tasks; kJ, kilojoules; LPA, low-intensity physical activity; MVPA, moderate-to-vigorous physical activity.

## Data Availability

The data presented in this study are available on request from the corresponding author.
